# Review on age-related differences in non-visual effects of light: melatonin suppression, circadian phase shift and pupillary light reflex in children to older adults

**DOI:** 10.1186/s40101-023-00328-1

**Published:** 2023-06-24

**Authors:** Taisuke Eto, Shigekazu Higuchi

**Affiliations:** 1 Research Fellow of the Japan Society for the Promotion of Science, Kodaira, Japan; 2 Department of Sleep-Wake Disorders, National Center of Neurology and Psychiatry, National Institute of Mental Health, Kodaira, Japan; 3 Department of Human Life Design and Science, Faculty of Design, Kyushu University, Fukuoka, Japan

**Keywords:** Aging, Non-image forming effect, Older adults, Children, Light, Crystalline lens, Pupil, Melatonin, Circadian rhythm, Pupillary light reflex

## Abstract

Physiological effects of light exposure in humans are diverse. Among them, the circadian rhythm phase shift effect in order to maintain a 24-h cycle of the biological clock is referred to as non-visual effects of light collectively with melatonin suppression and pupillary light reflex. The non-visual effects of light may differ depending on age, and clarifying age-related differences in the non-visual effects of light is important for providing appropriate light environments for people of different ages. Therefore, in various research fields, including physiological anthropology, many studies on the effects of age on non-visual functions have been carried out in older people, children and adolescents by comparing the effects with young adults. However, whether the non-visual effects of light vary depending on age and, if so, what factors contribute to the differences have remained unclear. In this review, results of past and recent studies on age-related differences in the non-visual effects of light are presented and discussed in order to provide clues for answering the question of whether non-visual effects of light actually vary depending on age. Some studies, especially studies focusing on older people, have shown age-related differences in non-visual functions including differences in melatonin suppression, circadian phase shift and pupillary light reflex, while other studies have shown no differences. Studies showing age-related differences in the non-visual effects of light have suspected senile constriction and crystalline lens opacity as factors contributing to the differences, while studies showing no age-related differences have suspected the presence of a compensatory mechanism. Some studies in children and adolescents have shown that children’s non-visual functions may be highly sensitive to light, but the studies comparing with other age groups seem to have been limited. In order to study age-related differences in non-visual effects in detail, comparative studies should be conducted using subjects having a wide range of ages and with as much control as possible for intensity, wavelength component, duration, circadian timing, illumination method of light exposure, and other factors (mydriasis or non-mydriasis, cataracts or not in the older adults, etc.).

## Background

Many living organisms, including humans, have evolved under sunlight, and information about the ambient environments that humans obtain through light and the physiological responses induced by light in humans are diverse. The light that enters the eye, passes through the pupil and crystalline lens, and reaches the retina is processed by two major pathways in the brain to induce physiological effects. The first is the visual effect that occurs when light information reaches the visual cortex, where it is processed to perceive brightness and color vision. Light information converted to neural signals by classical cones and rods is projected to the visual cortex via the thalamic lateral geniculate nucleus (LGN) using the optic tract. The other is processed in the pathway where the neural signals directly reach the suprachiasmatic nucleus (SCN) in the hypothalamus via the retinohypothalamic tract (RHT), which causes physiological effects to synchronize the biological clock (circadian clock), such as sleep/wake rhythms with the 24-h light/dark cycle associated with the earth’s rotation. These physiological effects of light are called non-visual (or non-image forming) effects, as distinguished from visual effects, and include light-induced melatonin suppression and pupillary light reflex in addition to the light entrainment effects of circadian rhythms.

Light performs a role as the strongest entrainment factor for circadian rhythms. It has been shown that bright light at night, such as artificial lighting, causes a delay and disruption of circadian rhythms and sleep [1] in both rural areas [2–4] and urban areas [5, 6], and is associated with various health problems [7]. It has been found that circadian rhythm phases are advanced by camp life without or with less access to artificial lighting in adults [8] and children [9]. These results suggest that artificial lighting at night causes delay in circadian rhythms in humans in modern society. Various factors have been shown to influence the non-visual effects of light. For example, the non-visual effects of light have been found to vary with differences in intensity [10–12], wavelength [13–15], exposure duration [16–18], and exposure circadian timing [19, 20]. The field of physiological anthropology, which focuses on environmental adaptability, also has a long history of research on the non-visual effects in light environments [21]. Physiological anthropology has also focused on variations in physiological responses from the perspective of environmental adaptability [22].

**Fig. 1:**
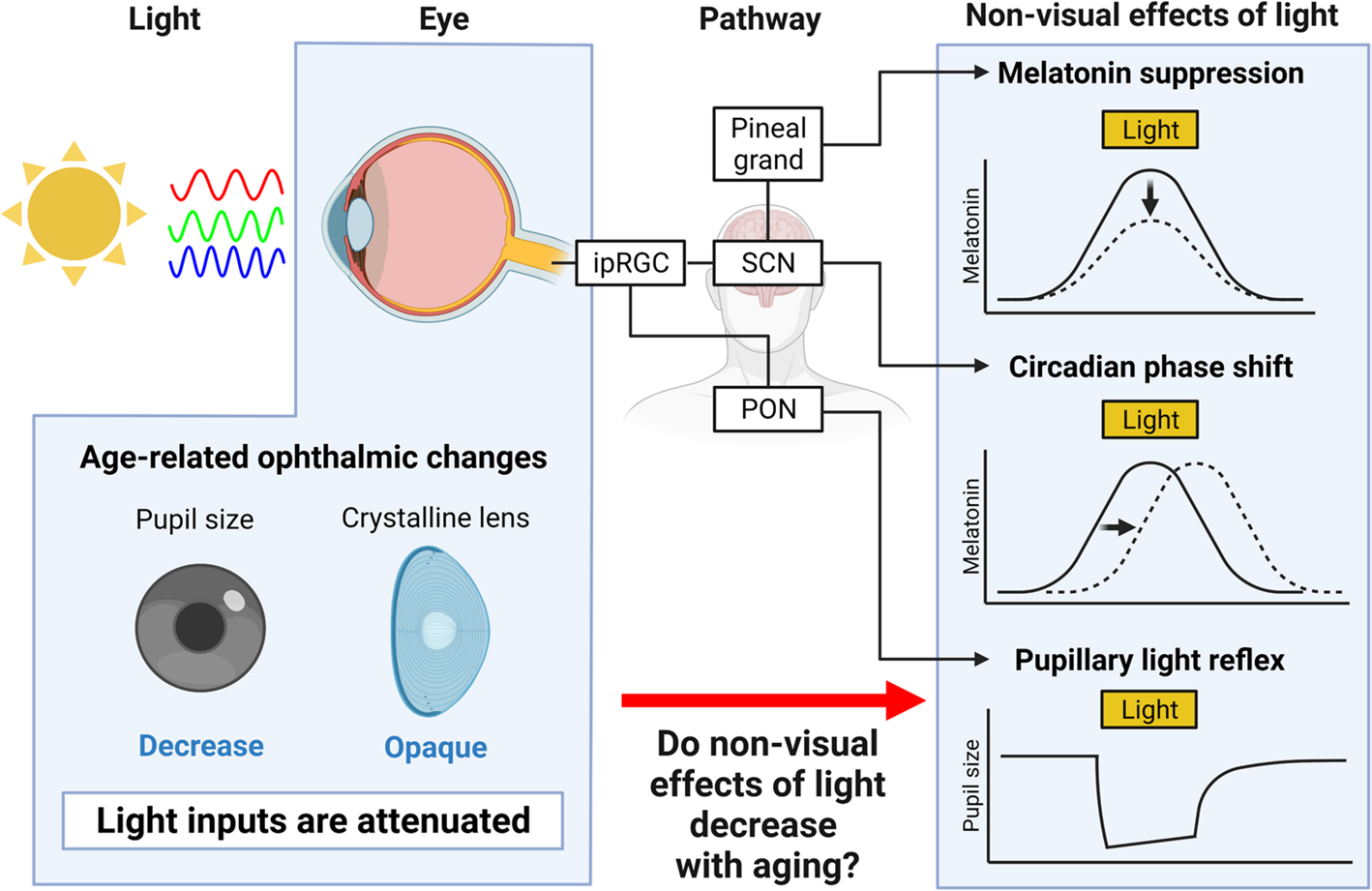
Conceptual scheme of age-related differences in non-visual effects of light (created with biorender.com)

The non-visual effects of light are modulated not only depending on light exposure conditions but also on factors on the human side. Recently, attention has been paid to individual differences in non-visual responses to light [23, 24]. While it has been reported that the non-visual effects of light vary depending on genetics [25, 26], season [27–29], ethnicity [30], and individual light exposure history [31–33], the most commonly studied factor is age [34]. In physiological anthropology, age-related differences in the non-visual effects of light are an important topic in terms of development [35] and aging [34]. Figure 1 shows that a conceptual scheme of age-related differences in the non-visual effects of light. This paper is a review of past studies and recent studies on age-related differences in light-induced melatonin suppression, light entrainment of circadian rhythms, and pupil light reflex.

## Age-related differences in melatonin suppression

Melatonin is a hormone that is biosynthesized in the pineal gland, and its production rhythm is regulated by the SCN, a central biological clock, via pathways of the paraventricular nucleus, pre-sympathetic ganglion neurons, superior cervical ganglion, and pineal gland [36]. Melatonin secretion shows a distinct circadian rhythm, with little secretion during the day, beginning at night, peaking at midnight and ending in the morning, making the secretory rhythm a quantitative indicator of the phase of the circadian rhythm. It is also known that melatonin secretion is acutely suppressed by light exposure [37]. Melatonin suppression is induced when incident light information from the eye reaches the SCN via retinal ganglion cells containing the photopigment melanopsin that contributes to non-visual effects (called mRGCs: melanopsin-containing retinal ganglion cells, or ipRGCs: intrinsically photosensitive retinal ganglion cells) and then the pineal gland. The degree of suppression of melatonin secretion is often used as an indicator of non-visual photosensitivity [10], and many studies have been conducted on age-related differences.

It is known that melatonin secretion itself also decreases with age [38, 39], and there are individual differences in melatonin secretion even in the same age group [40]. In studies in which light-induced melatonin suppression was examined, the rate of melatonin suppression was used with respect to an individual’s melatonin concentration measured in a dim-light environment [37] or pre-exposure melatonin concentration [41] to exclude the effects of individual differences in melatonin secretion. Therefore, differences in melatonin secretion due to aging are expected to have little effect on differences in melatonin suppression rates. However, it may be necessary to examine how the light-induced melatonin suppression affects physical and mental conditions not only in terms of the melatonin suppression but also in terms of differences in secretion [38, 39, 42].

### Melatonin suppression in the older adults

**Fig. 2:**
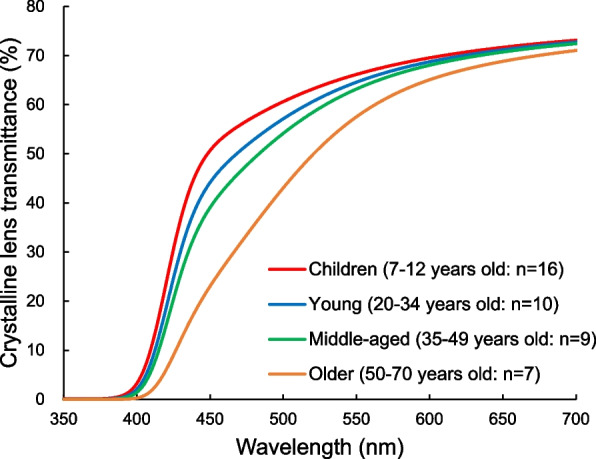
Crystalline lens transmittance spectra in vivo measured by Purkinje image-based system [50]. The lens transmittance spectrum in children is shown by a red line, that in young adults is shown by a blue line, that in middle-aged adults is shown by a green line, and that in the older adults is shown by an orange line. Depicted from data in Eto 2020 [50] and Eto 2021 [51]

Regarding age-related differences in melatonin suppression, Herljevic et al. first reported results of a comparison of melatonin suppression in young adults (mean age ± SD, 24 ± 3 years) and older adults (57 ± 5 years) in 2005 [43]. While there was no statistically significant difference in melatonin suppression between the young and the older adults after 30 min of exposure to green light (548 nm) (although suppression tended to be smaller in the older adults), the older adults had significantly lower melatonin suppression than the young adults when exposed to blue light (456 nm) for 30 min. As for the reason for the significant age-related difference only for exposure to blue light, Herljevic et al. mentioned the involvement of senile constriction of the pupil and age-related opacity of the crystalline lens. With aging, the pupil size diminishes [44, 45] and the crystalline lens becomes opaque (decrease in light transmittance) [46]. The decrease in light transmittance of the lens is particularly pronounced in the short-wavelength (blue) light range [47–49]. Figure 2 shows that the crystalline lens transmittance spectra in vivo measured by a Purkinje image-based system which was developed by authors [50] (depicted from data in Eto 2020 [50] and Eto 2021 [51]). As mentioned above, it is known that ipRGCs are the main contributors to the non-visual effects of light, including the melatonin suppression effects [52–54]. ipRGCs respond most strongly to blue light around 480 nm among visible light [52]. In other words, melatonin suppression effects in the older adults are thought to be weakened as a result of the attenuation of ipRGCs stimulation due to filtering of blue light caused by pupil diameter and lens transmittance reduction [55]. Attenuation of melatonin suppression in the older adults was also reported by Gabel et al. in 2017 [56]. In their study, young subjects (mean ± SE age: 24.96 ± 0.58 years) and older subjects (63.58 ± 1.27 years) were exposed to fluorescent light with an illuminance of 250 lx and color temperature of 2700 K (WL condition) and to fluorescent light with an illuminance of 250 lx and color temperature of 9000 K (BL condition) during sleep deprivation. The younger subjects showed significant suppression of melatonin secretion in both lighting conditions (pronounced in the high color temperature BL condition), whereas the older subjects showed no significant suppression in either lighting condition. A recent study reported by Chellappa et al. in 2021 also showed differences in melatonin suppression between older and younger subjects [41]. Chellappa et al. compared melatonin suppression in three lighting conditions differing only in color temperature (40 lx illuminance, 2500, 3000, and 6500 K color temperatures) in young subjects (22–29 years old, average age: 25.2 years) and older subjects (58–70 years old, average age: 63.6 years). The results showed that melatonin suppression occurred in both age groups under all light conditions but that melatonin suppression only in the young group was enhanced under the color temperature condition of 6500 K. The reason why the enhancement of melatonin suppression in high color temperature light (6500 K) observed in young subjects could not be confirmed in the older adult was thought to be because the blue light component in high color temperature light was attenuated in the older adults due to age-related lens opacity [41].

On the other hand, some studies have shown that melatonin suppression response is maintained in the older adults. In 2014, Najjar et al. obtained spectral sensitivity curves of melatonin suppression using nine different monochromatic lights and compared the curves in young subjects (mean ± SE age: 25.8 ± 0.73 years) and older subjects (59.4 ± 0.99 years) [57]. Their results showed that the peak wavelength of the spectral sensitivity curve was significantly shifted toward longer wavelengths in the older subjects but that there was no attenuation of melatonin suppression in the short-wavelength light region in the older subjects [57]. The shift of the peak wavelength toward longer wavelengths may be due to the effect of light transmittance of the crystalline lens, but the fact that there was no significant difference in melatonin suppression in the short-wavelength light between the older and young subjects does not support the results of the previous study by Herljevic et al. [43]. Najjar et al. suggested that differences in transmittance of the crystalline lens, exposure light intensity and exposure duration between those studies may have caused the discrepancy in results. As for the maintenance of the melatonin suppression response in older adults, they speculated that there might have been a compensatory function that compensated for the reduced light input to the retina. The light sensitivity of non-visual functions has been shown to be affected by recent changes in light history [31–33]. It has been reported that reduced daytime light exposure in winter enhances melatonin suppression [27]. It has also been shown that wearing contact lenses that block short-wavelength light from 30 min before a 2-h nocturnal light pulse until the end of the pulse attenuates melatonin suppression, whereas after wearing the contact lenses for 16 days, melatonin secretion is suppressed to the same degree as that in the control condition [58]. In other words, attenuation of retinal illuminance associated with changes in crystalline lens transmittance and pupil size in older adults can be viewed as changes in long-term light history, which may have resulted in increased (apparently maintained) light sensitivity as a physiological adaptation. On the other hand, Najjar et al. [57] performed mydriatic procedures (pupil being dilated) on their subjects, whereas the other studies [41, 56] on melatonin suppression in older adults did not, and this difference in methodology may also have led to discrepancies with the results of other studies.

### Melatonin suppression in pre-school children, school children and adolescents

As mentioned above, there have been many studies on age-related differences in the light-induced melatonin suppression that were conducted using older subjects. On the other hand, if pupil and crystalline lens characteristics affect age-related differences in the non-visual effects of light, the light-induced melatonin suppression would be expected to be stronger in populations with larger pupils and high crystalline lens transmittance (such as young, school-age and adolescent children) than in young adults.

**Fig. 3:**
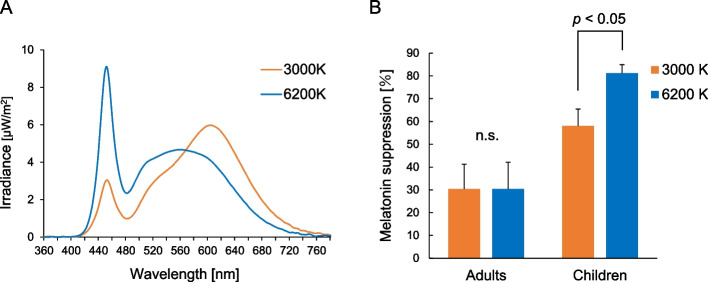
Spectral irradiance distributions of 3000 K and 6000 K lighting conditions (A) and melatonin suppression in adults and children under each lighting condition (B). Modified and adapted from Lee 2018 [61]

Some studies on light-induced melatonin suppression have also been conducted with children in childhood, early childhood, and adolescence. A study in young children (mean age: 4.3 ± 1.1 years) showed that melatonin suppression also occurs robustly in young children, although it is not clear whether there is childhood-specific light responsiveness because melatonin suppression in young children was not compared with that in other age groups [59]. In 2014, Higuchi et al. reported the results of a comparison of melatonin suppression rates in school children (8.6 ± 1.5 years old) and their parents (middle-aged: 41.2 ± 4.8 years old) [60]. The rate of melatonin suppression at habitual bedtime was approximately 1.9-times greater in the children than in the middle-aged subjects (children: 88.2%, middle-aged subjects: 46.3%) during exposure to white fluorescent light at an illuminance of 580 lx. Lee et al. in 2018 reported the results of a comparison of melatonin suppression in school children (8.9 ± 2.2 years) and middle-aged adults (41.7 ± 4.4 years) exposed to two LED lighting conditions with illuminance fixed at approximately 300 lx and differing only in color temperature (3000 and 6200 K) [61]. Melatonin suppression was significantly greater for the 6200 K lighting condition, which contains more blue components, than for the 3000 K lighting condition in children, whereas no significant difference between the color temperature conditions was observed in the middle-aged subjects (Fig. 3; Modified and adapted from Lee 2018 [61]). Enhanced melatonin suppression in response to high color temperature lighting was also found in a study on adolescents [62].

The enhanced melatonin suppression response in children compared to that in middle-aged people [60] and the enhanced melatonin suppression in response to high color temperature light only in children [61] were also thought to be due to age-related differences in pupil diameter and lens light transmittance. However, it has been difficult to investigate the relationship between optical characteristics of the eye and melatonin suppression because there is no established methodology for measuring crystalline lens transmittance (especially spectral transmittance), whereas measurement of pupil diameter is relatively easy. Recently, the authors developed a system that can easily measure spectral transmittance of the crystalline lens in vivo [50] and used it to investigate whether age-related differences in pupil size and lens transmittance are related to the difference between melatonin suppression in school children and that in middle-aged adults [51]. Melatonin suppression experiments were conducted in school children (9.6 ± 1.8 years old) and middle-aged adults (41.2 ± 2.5 years old) exposed to LED lighting with an illuminance of 300 lx and a color temperature of 6000 K, and pupil diameter during the light exposure and spectral transmission of the lens were also measured. The pupil diameter and spectral transmittance of the lens were used to calculate non-visual photoreception, which is an index of the influence of age-related ocular changes on the non-visual photopigment melanopsin, based on the method of Turner and Mainster [55], and the relationship between the age-related difference in non-visual photoreception and the age-related difference in melatonin suppression was evaluated. The results showed that relative values of non-visual photoreception and melatonin suppression in children to adults were 1.48 ± 0.08 (mean ± SE) and 1.52 ± 0.1, respectively, and both values were almost matched. This suggests that, at least between school children and middle-aged adults, age-related differences in pupil diameter and lens transmittance may influence the age-related differences in melatonin suppression effects.

On the other hand, Crowley et al. reported that the less sexually mature group (Pre/Mid-pubertal group) among school-age children to adolescents (9–16 years old) had stronger melatonin suppression responses than that in the more sexually mature group (Late/Post-pubertal group) [63]. Although it is difficult to generalize due to the lack of information on pupil diameter and crystalline lens transmittance, it is expected that there is no significant difference in pupil diameter or crystalline lens transmittance between the two groups, suggesting that factors other than ocular optical characteristics may be responsible for the age-related difference in melatonin suppression during the developmental period.

## Age-related differences in circadian phase shift

Circadian rhythm is the rhythm of an approximately 24-h cycle. Various circadian rhythms, such as sleep, body temperature and hormone secretion rhythms are regulated by the SCN, a central clock [64]. The endogenous circadian rhythm of the human body is considered to be slightly longer than 24 h [65], and the circadian rhythm cycle is synchronized to 24 h by the approach of ambient light information, such as sunlight, on the SCN. Specifically, the phase of the circadian rhythm can be advanced (become morningness) or delayed (become eveningness) depending on the timing of light exposure in a day. The phase response curve (PRC) indicates in which direction (advance or delay) the phase of the circadian rhythm shifts and how much it shifts depending on the timing of light exposure in a day [19, 20]. However, it has been reported that the phase response of the circadian rhythm varies with age, even when exposed to light at the same timing in the PRC.

### Circadian phase shift in the older adults

In 2007, Duffy et al. investigated the dose–response relationship between the illuminance of white fluorescent light (correlated color temperature: 4100 K) and the amount of circadian rhythm phase delay (IRC: illuminance response curve [66]) in older subjects aged 65 years or older (mean ± SD age: 68.3 ± 4.7 years) [67]. The rhythm of melatonin secretion, a marker of the circadian rhythm phase, was delayed by a maximum of about 3 h by light exposure during the biological night (a total of 6.5 h, from 30 min before habitual bedtime to 2 h before waking time). Duffy et al. compared the IRC in older subjects with that already reported in younger subjects aged 18 to 44 years (27.8 ± 8.9 years old) and found that although there was no age-related difference in the minimum (at 0 lx) and maximum (at ~ 10,000 lx) of phase delay, the illuminance that induced 50% of the maximum phase delay was higher in the older subjects than in the younger subjects (263 lx in the older subjects and 119 lx in the younger subjects) [10]. The delay of circadian rhythm phases at extremely low and high levels of illuminance is maintained in the older adults, in agreement with the results of previous studies [68, 69] and the results of a recent study [70]. Therefore, the light-induced shift effect of circadian rhythms is thought to be attenuated in the older adults in response to low to moderate illuminance (approximately 50 to 1000 lx). Duffy et al. suggested that senile constriction of the pupil and age-related opacity of the lens may be responsible for attenuation of the light-induced circadian rhythm shift in the older adults. As already mentioned, age-related reductions in pupil diameter and lens light transmittance in the blue light range are thought to attenuate the amount of ipRGCs stimulation in the older adults.

On the other hand, in 2009, Sletten et al. compared the amounts of phase advance in young subjects (23.0 ± 2.9 years) and older subjects (65.8 ± 5.0 years) during exposure to blue light (456 nm) and green light (548 nm) for 2 h in the morning (the timing of phase advance in PRC [20]), respectively [71]. Their results showed that the amount of phase advance tended to be greater in the younger subjects than in the older subjects when exposed to either blue or green light, but the difference was not statistically significant. Although the results of their study showing that these was no significant difference in the phase response between the older and younger subjects do not support the results of the study by Duffy et al. study mentioned above, Sletten et al. suggested that the differences in the type of light (white fluorescent light or monochromatic light) and intensity may have caused the difference in the results of the studies [71]. In addition to this, Sletten et al. performed mydriasis procedures on their subjects and this methodological difference may also have contributed to the differences between the results of the studies. More recently, in 2019, Scheuermaier et al. in the research group of Duffy et al. reported the results of an investigation of the amount of circadian phase delay in older subjects (58.3 ± 4.2 years old) exposed to approximately 270 lx white light showing a minimum of phase delay of 0.9 h and a maximum of 3.2 h among the older subjects, a large individual variation [66].

### Circadian phase shift in children and adolescents

Although, as far as the authors know, there has been no study in which the light responses of circadian rhythm phase in populations even younger than young adults were compared with these in other age groups, a PRC of adolescents aged 14 to 17 years (16.2 ± 1.0 years old) was shown by Crowley et al. in 2017 [72]. Crowley et al. found that the maximum values of phase advance and phase delay were larger in the PRC of adolescents that in the PRC of young adults reported by St. Hilaire et al. in 2012. However, they also noted that comparisons of the magnitude of phase shift between studies should be made with caution because of several methodological differences including differences in the intensity, duration and method of light exposure.

Few studies have experimentally evaluated light-induced circadian phase shifts in children. However, a field study has shown that camping life with much sunlight in the morning and little access to artificial lighting at night advances circadian phases in children (age range, 9–14 years) [9]. The results of this study were similar to those of Wright et al.’s study of the circadian phase shifts in adults (30.3 ± 8.5 years old) during camping life [8]. However, it is not easy to discuss age-related differences in light induced circadian phase shifts because these studies are not strictly environmentally controlled like laboratory experiments.

## Age-related differences in pupillary light reflex

Light information from ipRGCs is also transmitted to the pretectal olivary nucleus (PON), which is involved in the pupillary light reflex [54, 73]. The pupillary light reflex (PLR) refers to the increase or decrease in pupil size between approximately 2 and 8 mm in diameter to regulate the amount of light incident on the retina, and it is considered that vision over a wide range of brightness can be maintained by controlling light intensity [74]. The PLR is mediated not only by ipRGCs but also by rods and cones. The proportion of contribution among ipRGCs, rods and cones is known to depend on the intensity [75] and exposure time [76] of irradiated light. Specifically, the contribution of cones and rods is predominant for low-intensity incident light, whereas the contribution of ipRGCs is stronger for high-intensity incident light [75]. In addition, immediately after light exposure, the contribution of cones and rods is large, but the proportion of their contribution decreases as the exposure time increases from tens to hundreds of seconds, and the contribution of ipRGCs becomes dominant [76]. It is usually difficult to extract only the responsiveness of ipRGCs from PLR independently of the cone and rod responses, but the post-illumination pupil response (PIPR), in which the pupil remains contracted even after the end of light exposure (after the light stimulus is turned off), is known as a pupil response specific to ipRGCs [77]. PIPR has been used in various studies as an index to evaluate the light responsiveness of ipRGCs [78–81].

### PLR in the older adults

In contrast to the attenuation of light-induced melatonin suppression and circadian phase shift in the older adults, it has been reported that PLR maintains its responsiveness to light in the older adults [78, 82–84], and that although PLR is attenuated in the older adults, there is no wavelength dependence in the attenuation despite the fact that the short-wavelength light component that reaches the retina is decreased [85]. According to a report by Daneault et al. in 2012, when older subjects (61 ± 4.4 years old) and younger subjects (22.8 ± 4.0 years old) were exposed to blue light (480 nm) and green light (550 nm), there was no significant age-related difference in the pupil contraction rate regardless of the wavelength of light stimuli [82]. The studies by Kankipati et al. [78] and Adhikari et al. [84] on age-related changes in PIPR showed that there was no significant effect of age on PIPR, suggesting that light responsiveness of the pupil may be maintained even in older individuals [78, 84]. However, a study by Herbst et al. in 2012 showed that PIPR to blue light was positively correlated with age, suggesting that aging may rather enhance pupillary response [83]. Adhikari et al. discussed the discrepancy in their results as being related to the fact that cataract patients with lens opacity > grade 2 in LOCS III (lens opacities classification system III) [86] were excluded in their study [84]. The report by Herbst et al. did not mention any exclusion criteria for subjects with lens opacities and their study may have included older subjects with grade 3 or higher in LOCS III [83]. They hypothesized that the clouding of the lens in cataract patients may have increased the scattering component and stimulated more ipRGCs, thereby enhancing PIPR [83]. On the other hand, some studies have shown that PLR is attenuated in the older adults [85, 87]. Rukmini et al. in 2017 compared dose–response curves of the pupil constriction rate and light intensity when exposed to blue light (469 nm) and red light (631 nm) with various light intensities between young (21–30 years old) and older (50 + years old) subjects [85]. Their results showed that there was no wavelength dependence in age-related differences in pupil constriction, such as a particular attenuation for blue light, but there were age-related differences in pupil contraction rate, especially for high intensity light. Therefore, Rukmini et al. speculated that the decrease in pupil constriction, i.e., attenuation of the responsiveness of the PLR, in the older adults is due not to the effect of age-related changes in the crystalline lens but to aging changes in the autonomic nervous system [88] and retina [89]. Experimental parameters, such as light stimuli wavelength, intensity, and duration, in the studies mentioned in this section are summarized in Table 1.

**Table 1:** Comparisons of experimental parameters in studies on PLR or PIPR in older adults

Study	Subjects	Pupil condition	Metrics	Stimuli condition	Results
Daneaut et al. [82]. 2012	16 young adults (22.8 ± 4 years old), and 14 older adults (61 ± 4.4 years old) including subjects with level 2, 3 or 4 in LOCS III [86]	NOT dilated	Steady-state PLR (Averaged pupil size during 39 s of light exposure)	**Dark adaptation (0 lx):** 15 min before first stimulus; 2 min between stimuli **Wavelength:** Blue light (480 nm) and green light (550 nm) **Intensity:** Low: 7 × 10^12^ photons/cm^2^/s; Medium: 3 × 10^13^ photons/cm^2^/s; High: 10^14^ photons/cm^2^/s irradiance **Duration:** 45 s	There was no difference between age groups
Kankipati et al. [78]. 2010	37 normal subjects between ages of 19 and 80 years	Dilated	PIPR (Using average pupil size over a period of 30 s, starting 10 s after light offset)	**Dark adaptation:** None mentioned *Interval between red and blue stimuli was 5 min **Wavelength:** Blue light (470 nm) and red light (623 nm) **Intensity:** 13 log quanta/cm^2^/s (estimated retinal irradiance) **Duration:** 10 s	No significant correlation was found between PIPR and age
Adhikari et al. [84]. 2015	59 healthy subjects between ages of 21 and 70 years. All participants had a lens grading of ≤ 2 in LOCS III	Dilated and NOT dilated	PLR (Transient PLR, peak pupil constriction) and PIPR (6 s PIPR, Plateau PIPR, AUC early, AUC late)	**Dark adaptation (< 6 lx):** 10 min **Wavelength:** Blue light (448 nm) and red light (604 nm) **Intensity:** Blue: 14.5 log quanta/cm^2^/s; Red: 14.4 log quanta/cm^2^/s (corneal irradiance) **Duration:** 1 or 10 s	There is no effect of age
Herbst et al. [83]. 2012	44 healthy subjects aged between 26 and 68 years	NOT dilated	PLR (Maximal pupil contraction) and PIPR (Sustained pupil contraction, AUC early, AUC late)	**Dark adaptation (0 cd/m**^**2**^**):** 1 min after 4 min mesopic lighting condition (0.74 cd/m^2^) **Wavelength:** Blue light (470 nm) and red light (660 nm) **Intensity:** 3, 30, 100 and 300 cd/m^2^ (corresponding irradiance when intensity is 300 cd/m^2^: Blue 14.8 log photons/cm^2^/s; Red 14.9 log photons/cm^2^/s) **Duration:** 20 s	Sustained pupil contraction and AUC early correlated positively with age
Rukmini et al. [85] 2017	60 young normal adults aged 21—30 years and 54 older adults aged ≥ 50 years including mild cataracts and severe cataracts	NOT dilated	Dose–response curve of PLR	**Dark adaptation:** 1 min **Wavelength:** Blue light (469 nm) and red light (631 nm) **Intensity:** Gradually increase over 2 min from 6.8–13.8 log photons/cm^2^/s **Duration:** 2 min	Irrespective of wavelength, pupillary responses were reduced in older individuals and further attenuated by severe, but not mild cataract

As noted above, there is no unified view on whether PLR is attenuated in the older adults compared to that in young individuals. This may be related to the fact that different light intensities, wavelengths, and measurement indexes were used in studies, and the results can therefore not be directly compared. The results also seem to vary depending on whether cataract patients are included in the study [84, 85]. Recently, findings on PLR [85] and PIPR [90] in cataract patients have been accumulating, and further studies are needed to determine whether PLR is attenuated in the older adults, including the effects of cataracts.

### PLR in school children

Although there have been many studies in which PLR age-related differences were compared in the older and young adults, there have been few studies in children. We compared the spectral sensitivity of PLR between school children (9.9 ± 1.2 years old) and young adults (22.1 ± 1.8 years old) to determine whether the higher lens transmittance in children affects the age-related differences in PLR [91]. The results showed that the peak wavelength of the PLR spectral sensitivity curve tended to differ between children and young adults and that the peak wavelength shifts toward shorter wavelengths in children compared with that in young adults [91]. PIPR, on ipRGC-derived pupillary response, was measured for the first time in children (9.0 ± 1.8 years old) by Ostrin in 2018 [92]. That study showed that the ipRGC-derived pupillary response can be measured in children as robustly as in adults [80], for whom PIPR has been previously measured. However, comparisons with other age groups have not been made, and it is not known whether child-specific responsiveness exists in the PIPR.

## Conclusions

In this review, among the non-visual effects of light in humans, studies on age-related differences in light-induced melatonin suppression, circadian phase shift, and pupillary light reflex, including studies conducted by the authors, were reviewed. Whether or not there are age-related differences in any of the non-visual functions seems to be a matter of debate, since no unified view has been reached due to differences in experimental conditions and methodologies. In addition, as factors contributing to age-related differences in non-visual functions, pupil diameter and crystalline lens transmittance were mainly discussed in this paper, but since various other age-related factors such as differences in the number of retinal ganglion cells [93], volume of the SCN [94], peptide expression (vasoactive intestinal polypeptide (VIP) [95, 96]; arginine vasopressin (AVP) [97]), density of GABAergic synapses [98] and clock gene expression in the SCN [99, 100] as well as differences in ophthalmologic characteristics are thought to be involved (see review articles for details [101–104]), and the effects of growth and aging on non-visual functions are expected to be complex [34]. In order to study age-related differences in non-visual effects in detail, comparative studies should be conducted using subjects having a wide range of ages with as much control as possible for intensity, wavelength component, duration, circadian timing, illumination method of light exposure, and other factors (mydriasis or non-mydriasis, cataracts or not in the older adults, etc.). In addition to the cross-sectional studies described above, longitudinal studies (although not easy) are also necessary to better understand age-related changes in the non-visual effects of light. Naturally, individual differences exist in the aging process, and the results of longitudinal studies would contribute significantly to clarification of the development and aging process of non-visual functions.

It is important to note that even if there are age-related differences in one of the non-visual effects of light, it does not necessarily mean that there are age-related differences in other effects as well. For example, it has been suggested that the light-induced melatonin suppression effects are functionally separate from the circadian rhythm phase shift effects [105]. Additionally, there are several subtypes of ipRGCs, each with different photosensitivity [106, 107] and projection brain regions (although some overlap) [108–110], and each mediates different non-visual functions [111–113]. The studies on the subtypes of ipRGCs have predominantly focused on animal models, such as mice and rats. However, several subtypes of ipRGCs have also been identified in the human retina [114], and each subtype exhibits distinct sensitivities and responses to light [115]. The density of ipRGCs decreases with age, and it has been reported that their decrease causes disturbances in body temperature and locomotor activity in rats [116], but a study on the ipRGCs in the human retina has shown that the degree of decrease in the number of ipRGCs in the human retina appears to vary by subtype [117]. As we have discussed, the effects of aging may differ depending on the non-visual functions, such as circadian entrainment, melatonin suppression and PLR, and Daneault notes that since these non-visual responses are mediated, at least in part, by different ipRGCs populations, it is plausible that the effects of aging differ [82]. Therefore, age-related differences in each of these non-visual effects would need to be assessed independently and with attention to the presence of ipRGCs subtypes.

Furthermore, if there are age-related differences in the non-visual effects of light, further studies are needed to determine the extents to which these age-related differences contribute to age-related differences in circadian clock function and sleep. In particular, from the viewpoint of physiological anthropology, it is important to clarify the effects of artificial lighting at night on various aspects of human health such as sleep quality. In addition, individual differences [23, 24] as well as age-related differences in non-visual functions are an important topics as non-visual responses to light in individuals of the same age depending on genotype [25, 26], season [27–29], ethnicity [30], and individual light exposure history [31–33]. Therefore, accumulation of data regarding age-related and individual differences in the non-visual effects of light as well as factors contributing to the differences in various age groups from children to the older adults is important for designing and providing appropriate light environments, especially nighttime light environments, for humans, and further research is needed.

## Data Availability

Not applicable.

## List of abbreviations

LGN: Lateral geniculate nucleus
SCN: Suprachiasmatic nucleus
RHT: Retinohypothalamic tract
mRGCs: Melanopsin-containing retinal ganglion cells
ipRGCs: Intrinsically photosensitive retinal ganglion cells
PRC: Phase response curve
IRC: Illuminance response curve
PON: Pretectal olivary nucleus
PLR: Pupillary light reflex
PIPR: Post-illumination pupil response
LOCS III: Lens opacities classification system III
VIP: Vasoactive intestinal polypeptide
AVP: Arginine vasopressin
